# A Case of Monstrosity

**Published:** 1853

**Authors:** William H. Miller

**Affiliations:** Louisville


ARTICLE VII.—*A Case of Monstrosity.*—By William H. Miller, M. D., of Louisville.

Mrs. C., an Irishwoman, was seized with labor pains on the 13th of November, 1853. Prof. L. Rogers was summoned to attend the case, and found when the os uteris was dilated, that the shoulder was presenting. After encountering more than ordinary difficulty, in the attempt to deliver by turning, he had Prof. Miller called to his assistance. With some difficulty Prof. M. passed his hand into the cavity of the uterus, but was a good deal embarrassed to find the feet of the child. In the various movements of the head, a conical stump, with a moveable flap, resembling somewhat a foot, was encountered; but failing to find the feet, or anything by which turning could be effected, or extractive efforts applied, he withdrew his hand, and requested Prof R. to make the attempt also. Prof. R. discovered nothing in the shape of a lower member, except this same conical appendage just alluded to, which, it was finally concluded, might be a deformed lower extremity. Whereupon Prof. M. again introduced his hand, and taking hold of it, applied as much extractive force as it permitted. He found it very difficult to change the position, or make any impression upon the fetus, the hand easily slipping off, but by repeatedly renewing this hold, and exerting as much force as possible, he finally succeeded in drawing it down into the vagina. He was now enabled to get hold of a large part of the abnormal member, and ultimately to extract the body of the child. Prof. Rogers extracted the head, and delivered the placenta, &c.

